# Supplementary material for: Bayesian Optimization of Machine Learning Classification of Resting-State EEG Microstates in Schizophrenia: A Proof-of-Concept Preliminary Study Based on Secondary Analysis
Source: Brain Sci. 2022 Nov 4;12(11):1497. doi: 10.3390/brainsci12111497 (PMC9688063; doi:10.3390/brainsci12111497)
Supplement: Supplementary file 1 [file brainsci-12-01497-s001.zip › SupplementaryTables.pdf]

Table S1. Bayesian optimization workflow for the training dataset

1. For $t = 1,2, \ldots$

2. Maximize acquisition function (b) over f and find the new point as x_t

$$x_t = \text{argmax } b\,(x|DATA_{1:t-1}) \qquad (2)$$

3. Posterior distribution update

$$y_t = f(x_t)$$

$$DATA_{1:t} = \{DATA_{1:t-1}, (x_t, y_t)\} \qquad (3)$$

4. End For.

Table S2. Hyperparameters of best-fitted ML models based on the importance score of the microstate features.

Number of microstate features	Best-fitted model	Model parameters
1	SVM	Kernel [Gaussian], Scale [0.017]
2	Ensemble	Method [LogitBoost], Num of cycles trained [300]
3	SVM	Kernel [Gaussian], Scale [0.680]
4	Tree	Number of nodes [105]
5	Ensemble	Method [LogitBoost], Num of cycles trained [300]
6	Ensemble	Method [LogitBoost], Num of cycles trained [299]
7	Ensemble	Method [LogitBoost], Num of cycles trained [218]
8	Ensemble	Method [LogitBoost], Num of cycles trained [299]
9	Ensemble	Method [LogitBoost], Num of cycles trained [200]
10	Ensemble	Method [LogitBoost], Num of cycles trained [299]
11	Ensemble	Method [LogitBoost], Num of cycles trained [299]
12	Ensemble	Method [LogitBoost], Num of cycles trained [238]
13	Ensemble	Method [LogitBoost], Num of cycles trained [204]
14	Ensemble	Method [LogitBoost], Num of cycles trained [299]
15	Ensemble	Method [LogitBoost], Num of cycles trained [299]
16	Ensemble	Method [LogitBoost], Num of cycles trained [202]
17	Ensemble	Method [LogitBoost], Num of cycles trained [298]
18	Ensemble	Method [LogitBoost], Num of cycles trained [226]
19	SVM	Kernel [Gaussian], Scale [74.76]
20	Ensemble	Method [LogitBoost], Num of cycles trained [229]

Table S3. Output measures of the subject-independent best-fitted ML models.

Leave out subject EEG segments [Test set data]	Best-fitted model	Output measures				Model parameters
		Sensitivity (%)	Specificity (%)	Acc (%)	AUC	
**HC[S1],SZ[S1]**	**Ensemble**	76.97	95.16	84.44	0.86	Method [LogitBoost], Num of cycles trained [298]
HC[S2],SZ[S2]	Ensemble	90.23	91.53	90.88	0.91	Method [LogitBoost], Num of cycles trained [237]
HC[S3],SZ[S3]	Neural network	81.82	100.00	90.09	0.91	Method [net], Activations [relu], Layer sizes [241,70,3]
HC[S4],SZ[S4]	Ensemble	86.67	88.95	87.89	0.88	Method [LogitBoost], Num of cycles trained [242]
HC[S5],SZ[S5]	Neural network	93.18	94.37	93.71	0.94	Method [net], Activations [relu], Layer sizes [253,31,7]

HC[S6],SZ[S6]	Ensemble	94.05	83.61	89.90	0.89	Method [LogitBoost], Num of cycles trained [285]
HC[S7],SZ[S7]	Ensemble	96.57	75.45	84.81	0.86	Method [LogitBoost], Num of cycles trained [275]
HC[S8],SZ[S8]	Neural network	83.54	88.65	85.90	0.86	Method [net], Activations [tanh], Layer sizes [181,52,7]
HC[S9],SZ[S9]	SVM	95.35	95.14	95.24	0.95	Kernel [Gaussian], Scale [2.47]
HC[S10],SZ[S10]	Neural network	94.42	98.54	95.95	0.96	Method [net], Activations [relu], Layer sizes [61,4,3]
HC[S11],SZ[S11]	SVM	100.00	92.52	95.99	0.96	Kernel [Gaussian], Scale [5.48]
HC[S12],SZ[S12]	SVM	95.83	96.30	96.08	0.96	Kernel [Gaussian], Scale [2.17]
HC[S13],SZ[S13]	Ensemble	97.75	89.39	93.56	0.94	Method [LogitBoost], Num of cycles trained [294]
HC[S14],SZ[S14]	Neural network	95.05	86.92	89.73	0.91	Method [net], Activations [relu], Layer sizes [87,17,48]
Mean ± SD		91.53 ± 6.76	91.18 ± 6.44	91.01 ± 4.18	0.91 ± 0.038	Suggested models [Ensemble, SVM, and Neural network]
