# Supplementary figures and images for: Bayesian Optimization of Machine Learning Classification of Resting-State EEG Microstates in Schizophrenia: A Proof-of-Concept Preliminary Study Based on Secondary Analysis
Source: Brain Sci. 2022 Nov 4;12(11):1497. doi: 10.3390/brainsci12111497 (PMC9688063; doi:10.3390/brainsci12111497)

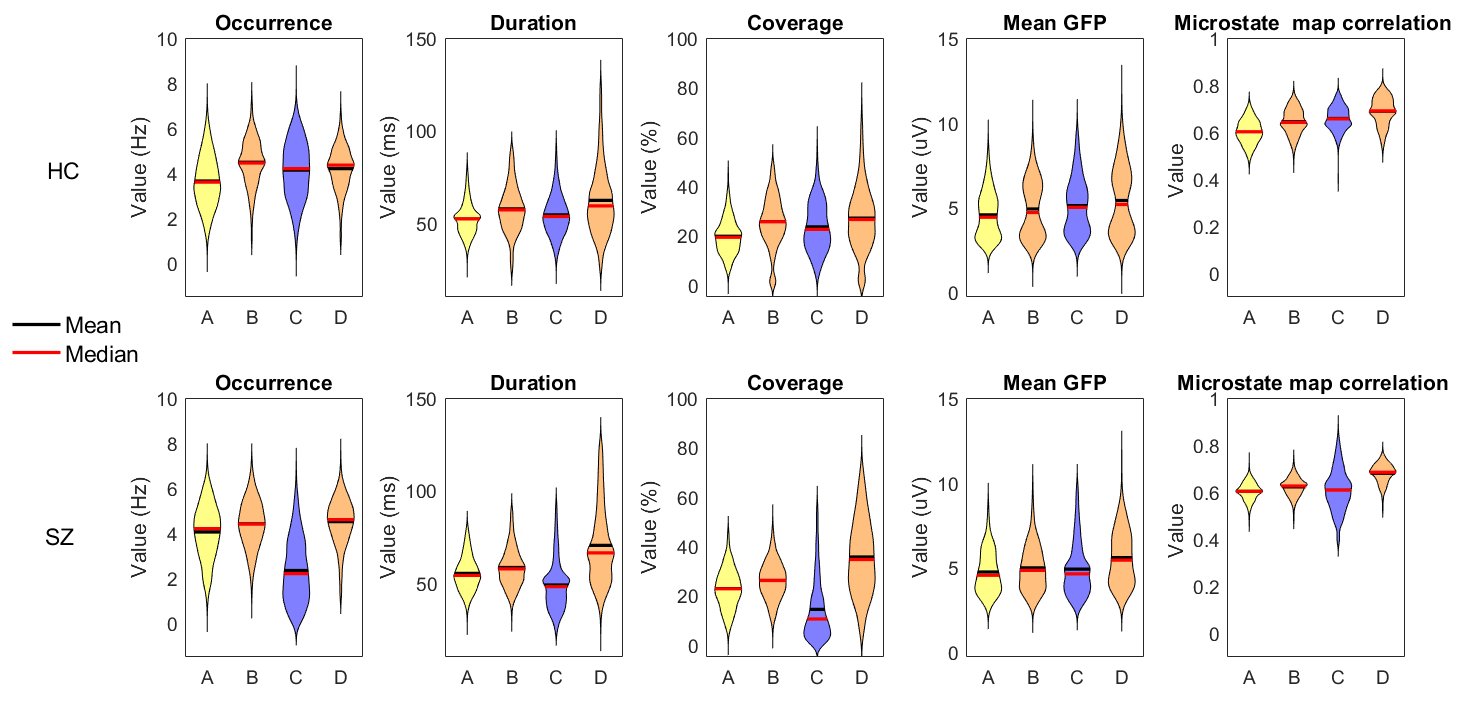

Supplement: Supplementary file 1 [file brainsci-12-01497-s001.zip › Figure S1.tiff]

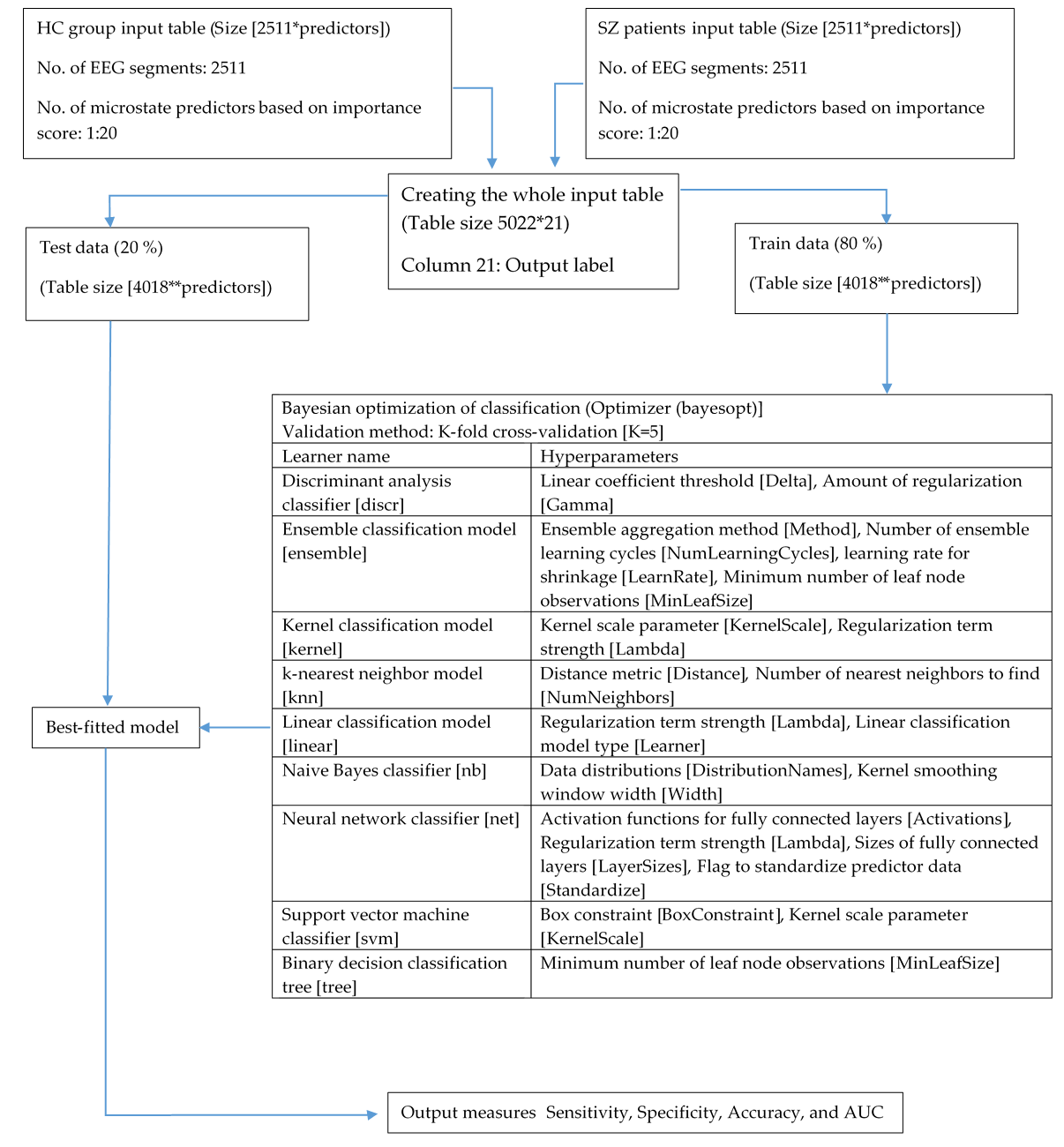

Supplement: Supplementary file 1 [file brainsci-12-01497-s001.zip › Figure S2.tiff]

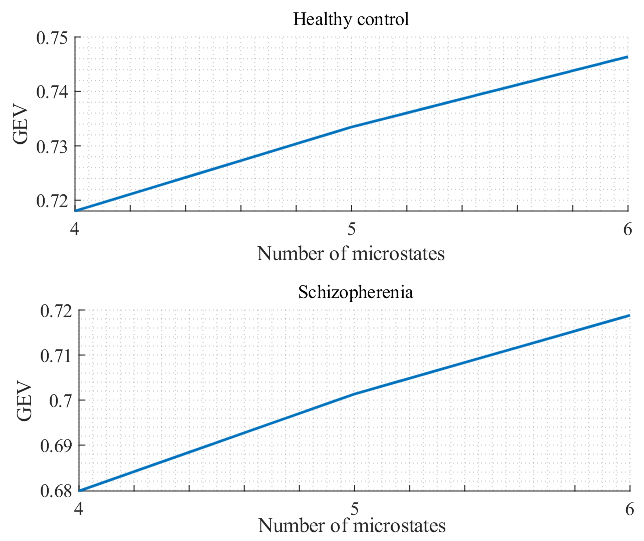

Supplement: Supplementary file 1 [file brainsci-12-01497-s001.zip › Figure S7.tiff]
